# Superiority of native seed core microbiomes in the suppression of bacterial wilt disease

**DOI:** 10.3389/fmicb.2024.1506059

**Published:** 2025-01-15

**Authors:** Yanling Dong, Jie Gong, Lei Yang, Qipeng Jiang, Chengzhi Wen, Jidan Zhang, Ruiyu Yang, Yao Wang, Yuhao Dai, Gui Gao, Shili Li, Yi Cao, Wei Ding

**Affiliations:** ^1^College of Plant Protection, Southwest University, Chongqing, China; ^2^Agricultural and Rural Affairs Committee of Fuling District, Chongqing, China; ^3^China Tobacco Hunan Industrial Co., Ltd., Changsha, China; ^4^Qianxinan Tobacco Branch of Guizhou Tobacco Company, Xingyi, China; ^5^Guizhou Academy of Tobacco Science, Guiyang, China

**Keywords:** endophytic bacteria, *Paenibacillus*, resistant varieties, susceptible varieties, tobacco bacterial wilt

## Abstract

**Introduction:**

Native endophytic microorganisms in tobacco seeds are closely related to their resistance to *Ralstonia solanacearum* (*R. solanacearum*) infections. However, the role of the native seed core microbiome in the suppression of bacterial wilt disease (BWD) remains underexplored.

**Methods:**

The characteristics of endophytic bacterial communities in both resistant and susceptible tobacco varieties were characterized using high-throughput sequencing technology.

**Results:**

This study found *Paenibacillus* as a potential microbial antagonist against BWD based on its significantly greater presence in BWD-resistant tobacco varieties, with a relative abundance that was 83.10% greater in the seeds of resistant tobacco than in those of susceptible varieties. Furthermore, a *Paenibacillus* strain identified as *Paenibacillus odorifer* 6036-R2A-26 (*P. odorifer* 26) was isolated from the seeds of the resistant variety. Following irrigation treatment with *P. odorifer* 26, the BWD index was reduced by 51.08%. Additionally, this strain exhibited significant growth-promoting effects on tobacco. It significantly increased the fresh weight of the tobacco plants by 30.26% in terms of aboveground weight, 37.75% in terms of underground weight, and 33.97% in terms of aboveground dry weight. This study highlights the critical role of *Paenibacillus* in tobacco seeds in the suppression of BWD, which may result from its antagonistic and growth-promoting properties.

**Discussion:**

The results of this study revealed differences in the structural characteristics of endophytic bacterial communities between resistant and susceptible tobacco varieties, with groups such as *Paenibacillus* potentially playing significant roles in resisting BWD. These findings highlight the superiority of seed endophytic microorganisms. In the context of declining plant disease resistance and the spread of bacterial wilt, core endophytic microorganisms in seeds may emerge as a viable option for enhancing the productivity of agricultural ecosystems.

## 1 Introduction

Endophytic bacteria are an essential component of plant microecological systems (Verma et al., 2021). As microorganisms that colonize the internal tissues or organs of plants, their presence usually does not have a negative effect on the host (Lopez et al., 2012). Numerous studies have revealed beneficial relationships between endophytic bacteria and their hosts (Vandana et al., 2021), such as inhibiting diseases (Ritpitakphong et al., 2016), stimulating plant immune systems and inducing systemic resistance (Van der Ent et al., 2009), helping plants obtain nutrients (Van Der Heijden et al., 2016), increasing resistance to abiotic stresses (Rolli et al., 2015), adapting to environmental changes, and other beneficial effects (Haney et al., 2015).

As storage vessels for beneficial microorganisms (Rahman et al., 2018), plant seeds can increase plant adaptability by improving seed quality and protecting seedlings from pathogen infection (Adam et al., 2018; Johnston-Monje et al., 2016; Shade et al., 2017). Seed endophytic bacteria play roles in promoting plant growth and defending against environmental stress (Khalaf and Raizada, 2016), mainly manifesting as follows: seed endophytic bacteria promote seed germination and maintain plant health by providing benefits to offspring plants through vertical transmission, thus maintaining beneficial connections between multiple generations of plants (Chee-Sanford et al., 2006; Truyens et al., 2015). Moreover, seed-associated microorganisms play important roles in nutrient uptake and reducing abiotic and biotic stresses. Bacteria isolated from plant seeds have functions such as phosphorus solubilization, nitrogen fixation, production of growth hormones, and synthesis of antimicrobial compounds (Zhang et al., 2022). There is also a significant amount of research demonstrating the inhibitory effects of seed-borne microorganisms on pathogens (Verma et al., 2018). Current research on endophytic bacteria in plant seeds has focused mainly on crops such as rice, wheat, peanuts, medicinal plants, and pumpkins, concentrating on aspects such as the community composition of seed endophytic bacteria, their biocontrol potential, and vertical transmission (Chen et al., 2018; Herrera et al., 2016; Li et al., 2019). However, relatively little research has been conducted on tobacco seed endophytic bacteria.

Bacterial wilt disease (BWD), a major devastating disease caused by *R. solanacearum*, leads to serious economic losses worldwide (Abo-Elyousr et al., 2019; Mansfield et al., 2012), especially in warm–temperate or tropical and subtropical areas (Hayward, 1991; Jiang et al., 2017; Lai et al., 2021). Many studies have shown that the occurrence of plant wilt disease is closely related to the presence of symbiotic microorganisms, such as rhizosphere microorganisms, root surface microorganisms, and endophytic microorganisms. Seeds, as important media for plant resistance, play a significant role in plant growth. However, research on the correlation between endophytic microorganisms in seeds and their disease resistance is still relatively lacking. The structural characteristics of the core microbial community in seeds need further clarification, and the mechanisms of disease resistance mediated by seed microorganisms require further investigation. In recent years, the use of effective biocontrol strains has become popular and has proven to be a promising strategy for the management of plant diseases (Spadaro and Gullino, 2004).

This study used tobacco seeds with different BWD resistance phenotypes as research materials. We characterized the endophytic bacterial communities in seeds of different resistant varieties and isolated and screened antagonistic endophytic bacteria from seeds of tobacco varieties exhibiting resistance phenotypes. We hypothesized that the endophytic bacterial community in seeds contributes to tobacco resistance to BWD and that specific microbiomes play critical antagonistic roles in managing *R. solanacearum*. The aims of this study were to (1) characterize the bacterial communities in tobacco seeds of varieties that are resistant and susceptible to BWD and (2) identify potential antagonists for BWD management. The results of this study provide a theoretical basis and material support for innovative BWD management strategies.

## 2 Materials and methods

### 2.1 Sample collection

This study employed four tobacco varieties, 6036, G3, Honghua Dajinyuan (HD), and Yunyan 87 (YY87), which were all planted in May 2020 at the Cha Shu Ping experimental area in Runxi township, Pengshui County, Chongqing City (elevation 1,360 m, 29°7′43″N, 107°56′38″E). The pathogen was isolated from diseased tobacco plants in the Baiguo Ping village, Pengshui County, Chongqing City (elevation 1,210 m, 29°8′12″N, 107°56′31″E) and was identified as the highly pathogenic *R. solanacearum* strain CQPS-1 (Liu et al., 2017).

### 2.2 Evaluation of tobacco variety resistance

#### 2.2.1 Disease incidence in fields

Flat plots with perennial BWDs were selected, three replicates were set for each variety, and ~1,200 tobacco plants were planted. The incidence of BWD in each replicate was calculated every 5 days starting at the onset of the disease (Liu et al., 2016).

#### 2.2.2 Indoor pot experiments

The pot experiments involved floating seedling cultivation of different tobacco varieties, which were subsequently grown until four leaves and one heart were developed for later use. Root inoculation was performed for each tobacco seedling with 10 mL of a CQPS-1 bacterial suspension at a concentration of 10^8^ CFU/mL. Each treatment was repeated three times, with eight seedlings per repetition. For the pot experiment, a systematic investigation of disease occurrence in different varieties began at the early stage of infection. Surveys were conducted every 2 days until the susceptible varieties reached the end of disease progression. Disease indices and survival curves were calculated (Zhang et al., 2023).

### 2.3 DNA extraction

Approximately 5 g of surface-sterilized seeds was ground into a homogenate with the addition of liquid nitrogen. DNA was extracted using the FastDNA™ SPIN Kit according to the manufacturer's instructions. After quality inspection, PCR amplification was conducted on the V5–V7 variable region of the 16S rDNA of the endophytic bacteria in the seeds (Zhang et al., 2022).

The PCR system was constructed as follows: 4 μL of 5 × FastPfu Buffer, 2 μL of 2.5 mM dNTPs, 0.8 μL of forward primer (5 μM), 0.8 μL of reverse primer (5 μM), 0.4 μL of FastPfu Polymerase, 0.2 μL of BSA, 10 ng of template DNA, and ddH_2_O to a final volume of 20 μL.

The amplification procedure involved two rounds of nested PCR (Table 1): the first round utilized the primers 799F and 1392R, and the extracted DNA was used as the template to amplify the V5–V8 variable region of the endophytic bacteria 16S rDNA. The conditions were as follows: 95°C for 3 min of initial denaturation, followed by 27 cycles of 30 s at 95°C for denaturation, 30 s at 55°C for annealing, and 45 s at 72°C for extension, with a final extension at 72°C for 10 min. In the second round, the amplification product from the first round served as the DNA template, and primers 799F and 1193R were used to amplify the V5–V7 variable region of the 16S rDNA, with 13 cycles and the same conditions as those used in the first round (Shi et al., 2023). Once the amplification products passed quality inspection, they were sent to Shanghai Meiji Biotechnology Co., Ltd., for Illumina MiSeq sequencing.

**Table 1:** Sequencing primer sequences for amplifying bacterial amplicons.

**Primer**	**Sequence (5^′^-3^′^)**	**Amplification area**
799F	AACMGGATTAGATACCCKG	V5–V8 variable region
1392R	ACGGGCGGTGTGTRC	
799F	AACMGGATTAGATACCCKG	V5–V7 variable region
1193R	ACGTCATCCCCACCTTCC	

### 2.4 Isolation and identification of endophytic antagonistic bacteria from seeds

#### 2.4.1 Isolation and purification of endophytic bacteria

Using the dilution plating method, 2 g of surface-sterilized seeds from different tobacco varieties were finely ground in a mortar and then mixed with 8 mL of sterile water. After allowing the mixture to stand for 5 min, 100 μL of the supernatant was taken and diluted with 900 μL of sterile water, which was designated a 10^−2^ dilution. This dilution process was continued to achieve further dilutions of 10^−3^, 10^−4^, and 10^−5^. For each dilution, 100 μL of the diluted solution was spread onto nutrient agar (NA), tryptone soy agar (TSA), Reasoner's 2A agar (R2A), or King's B medium (KB) plates, and glass beads were used for even distribution. Three replicates were set up for each concentration and plate type, and the plates were inverted and incubated at 30°C for 2–5 days while bacterial growth was regularly observed. Based on differences in colony morphology, color, and transparency, different representative strains were selected and named according to their format (variety–culture medium–number). Single colonies were then picked from the corresponding plates and streaked for purification 3–4 times until the colonies on the plates were uniform and free of contaminants, indicating that the purification process was complete (Li et al., 2019).

#### 2.4.2 Screening of antagonistic bacterial strains

The purified bacteria were preliminarily screened for antagonistic strains using the plate inhibition activity screening method. First, a single colony was picked and inoculated onto the center of an NA plate, and this process was repeated 3 times for each strain. The plates were incubated upside down at 30°C overnight. Using a sterile sprayer, an OD600 nm = 0.1 (10^8^ CFU/mL) suspension of pathogenic bacteria was evenly sprayed onto NA plates. After incubation at 30°C for 24 h, the diameter of the inhibition zone was measured using the cross-streak method (Tao et al., 2024). The strains with larger inhibition zones were selected for subculture, and their inhibitory stability was repeatedly tested. Ultimately, potential antagonistic bacteria with good and stable plate inhibition effects were identified. The strains with strong antibacterial activity selected from the screening plates were used for a preliminary evaluation of their pot culture effects.

#### 2.4.3 Molecular identification of isolates

A single colony was picked and transferred to LB medium and then incubated at 30°C and 180 rpm for 12–14 h. The bacterial cells were collected by centrifugation, and DNA was extracted using a bacterial DNA extraction kit. The extracted total DNA was used as a template for bacterial 16S rDNA amplification by PCR with the universal primers 27F (5′-AGAGTTTGATCCTGGCTCAG-3′) and 1429R (5′-TACGGCTACCTTGTTACGACTT-3′). The reaction system was 25 μL, to which 1.0 μL of template DNA, 1.0 μL of each of the upstream and downstream primers, 12.5 μL of 2 × Taq Master Mix and 9.5 μL of ddH_2_O were added. After PCR amplification, the products were checked for quality by 1% agarose gel electrophoresis and then sent to BGI Genomics for sequencing. The sequencing results were compared for homology using the National Center for Biotechnology Information (NCBI) database, and a phylogenetic tree was constructed using the neighbor-joining (NJ) method with bootstrap analysis (1,000 replicates) in MEGA X software (Haruna et al., 2021).

### 2.5 Evaluation of biocontrol efficacy

Single colonies of potential antagonistic bacteria were transferred to LB broth for shaking culture, and single colonies of the target pathogen were transferred to B liquid medium for shaking culture. Both cultures were shaken until the optical density at 600 nm reached 0.8–1.0 and were then diluted with sterile water to an OD_600_ of 0.1 (equivalent to 10^8^ CFU/mL) for later use. First, potential antagonistic bacterial fermentation broth was used for the root irrigation treatment (10 mL per plant at a concentration of 10^8^ CFU/mL). Three days after inoculation, the plants were root irrigated with the same concentration of the pathogenic bacteria (10 mL per plant at 10^8^ CFU/mL). The plants were then placed in a greenhouse at a temperature of 30°C, a relative humidity of 75%, and a light–dark cycle of 14/10 h. Each treatment was replicated twice, with 8 tobacco seedlings in each replicate. The onset of BWD was monitored from the initial stages, with observations conducted every 2 days using the same disease assessment method.

### 2.6 Measurement of tobacco seedling biomass

The experiment involved root irrigation using the bacterial fermentation liquid *P. odorifer* 26 at a concentration of 108 CFU/mL, with 10 mL applied per plant. Each treatment was conducted with three replicates, and each replicate consisted of five plants. The root irrigation was repeated every 7 days, utilizing the same volume, for a total of three applications. Ten days after the final inoculation, the biomass of tobacco seedlings from the different treatment groups was assessed. Initially, the root substrate was gently shaken off, the remaining substrate was washed away with clean water, and the surface moisture of the plants was removed with absorbent paper. Measurements were then taken for root length, aboveground fresh weight, and underground fresh weight. The samples were dried first by heating at 105°C for 15 min to kill the material, followed by drying at 75°C for 4 h, after which the aboveground dry weight and underground dry weight were recorded.

### 2.7 Statistical analyses

Excel 2016 was used to organize and summarize the data, analysis of variance and significance testing (*P* < 0.05) were performed using IBM SPSS Statistics 23, and Origin 2017 and GraphPad Prism 9 were used for data visualization.

Alpha diversity analysis was conducted based on the alpha diversity index. Analysis of similarity (Adonis) and permutational multivariate analysis of variance (PERMANOVA) were performed to evaluate significant differences in microbial community composition among seeds of different tobacco varieties. Linear discriminant analysis (LDA) coupled with effect size (LEfSe) is an algorithm that identifies features (i.e., genes, pathways, or taxa) characterizing differences between two or more biological conditions. Here, LEfSe was used to identify rhizobacterial taxa with significant differences in relative abundance between monocrop and intercropped peanut root systems as potential biomarkers (Li et al., 2023).

## 3 Results

### 3.1 Different tobacco varieties exhibit varying resistance to BWD

The manifestations of BWD in the middle and late stages of the field trials are shown in Supplementary Figure S1. We found that the incidence and severity indices of all tobacco varieties gradually increased over time, but the incidence and severity indices of HD and YY87 were always greater than those of 6036 and G3. The incidence and severity indices of the different varieties of tobacco were ranked as follows: 6036 > G3 > YY87 > HD.

**Figure 1:**
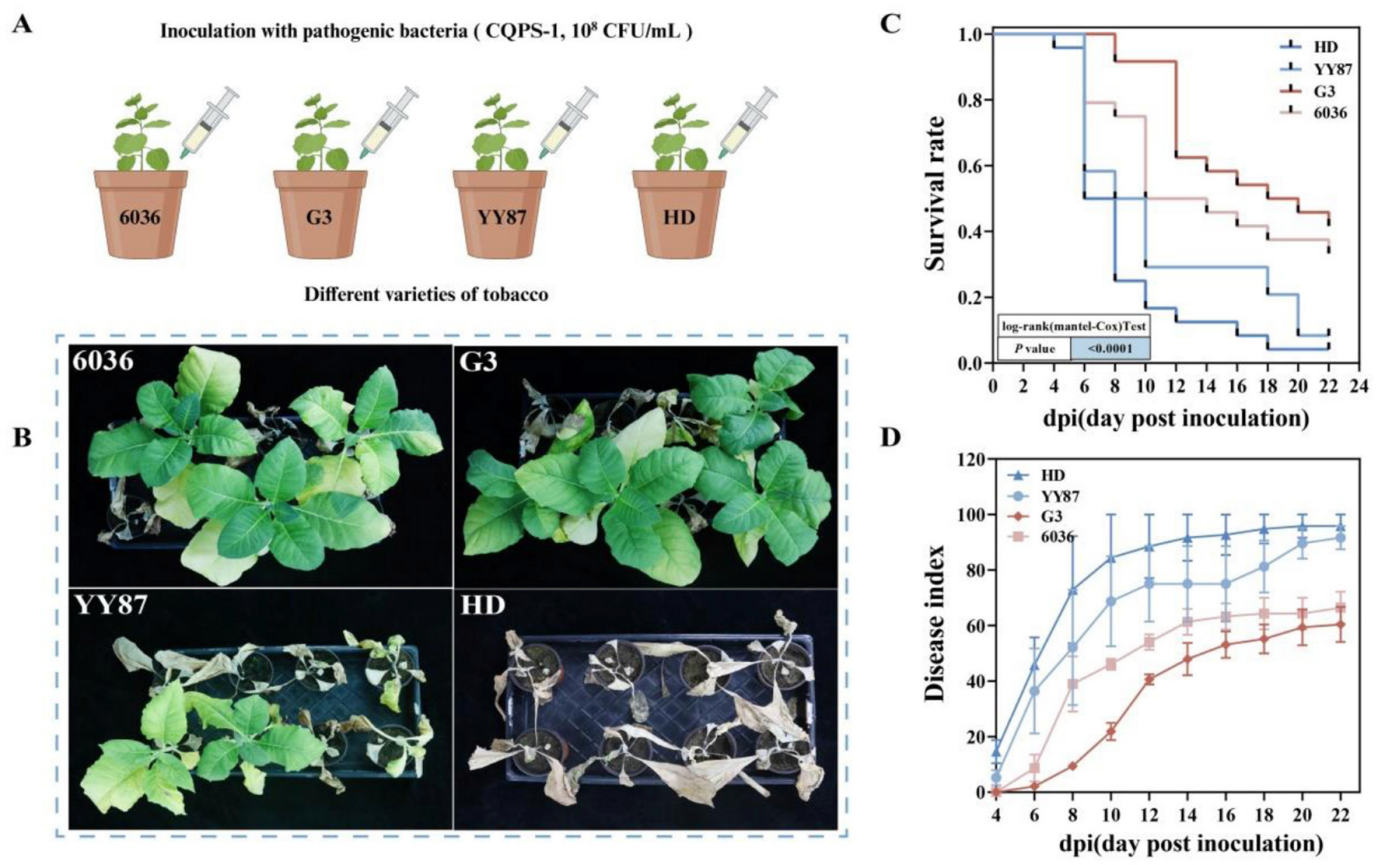
Assessment of disease resistance in different tobacco varieties. **(A)** Flowchart of the experimental methods. **(B)** Growth images of tobacco plants on the 22nd day after infection in the indoor pot experiment. **(C)** Survival curve of the disease resistance assessment in the indoor pot experiment. **(D)** Disease indices of different tobacco varieties in the indoor pot experiment.

The results of the indoor pot experiments were similar to those of the field experiments (Figure 1). As shown in Figure 1B, 6036 and G3 had strong resistance to BWD, whereas YY87 and HD had weak resistance to BWD. As shown in Figure 1C, in the late stage of infection, the survival rates for HD and YY87 were 4.2% and 12.5%, respectively, which were significantly lower than the survival rates of varieties 6036 and G3, which were 33.3% and 37.5%, respectively. At 22 days postinoculation, which marks the late stage of the disease, the average severity indices for the HD and YY87 varieties were 95.83 and 91.67, respectively, which were significantly higher than the average severity indices of the 6036 and G3 varieties, which were 66.44 and 60.42, respectively (Figure 1D). Based on the analysis of the disease indices and survival curves throughout the investigation period, the degree of resistance to BWD in the tobacco varieties ranked from high to low as follows: G3 > 6036 > YY87 > HD. Based on both the indoor pot and field experiments, varieties 6036 and G3 were preliminarily identified as resistant to disease, whereas YY87 and HD were identified as susceptible.

### 3.2 Differential composition of endophytic bacterial community structure in seeds of tobacco varieties with varying resistance levels

**Figure 2:**
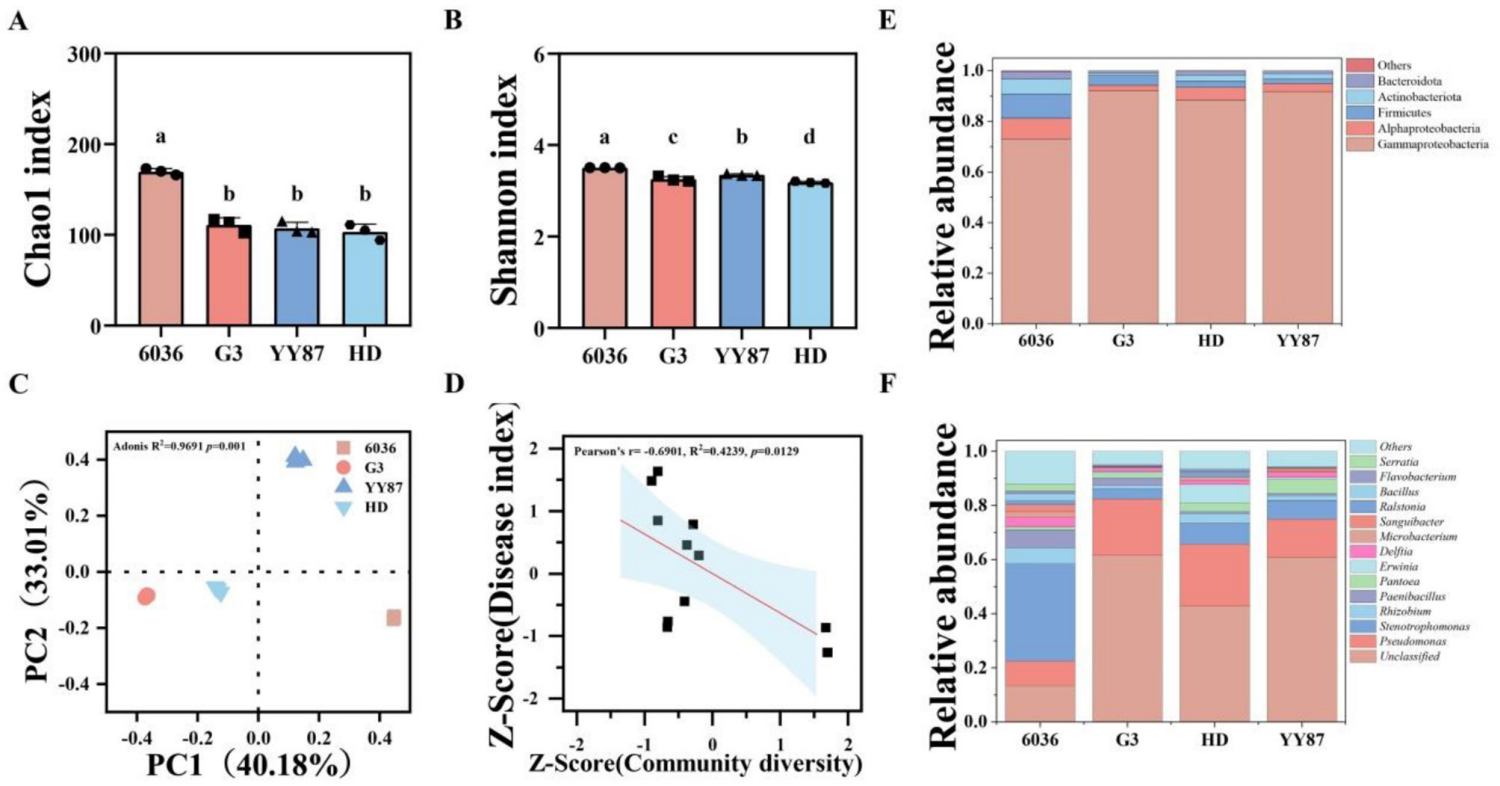
Diversity and composition analysis of endophytic bacterial communities in seeds of different resistant tobacco varieties. **(A)** Chao1 index; **(B)** Shannon index; **(C)** PCoA; **(D)** linear fit of disease index and diversity; **(E)** species abundance at the phylum level; **(F)** species abundance at the genus level. Different letters indicate significant difference at 0.05 level among different treatments.

The results of the alpha diversity analysis of the different varieties are shown in Figures 2A, B. The Chao1 and Shannon indices of the resistant variety 6036 were significantly greater than those of the susceptible varieties YY87 and HD. The Shannon index of the resistant variety G3 was significantly greater than that of the susceptible variety HD. The results of the alpha diversity analysis indicate that the resistant variety 6036 had a significantly greater observed species count and microbial community richness than the susceptible varieties. The results of the beta diversity analysis of the endophytic bacterial communities in the seeds of different varieties are shown in Figure 2C. The principal coordinate analysis (PCoA) results revealed that the *R* value from the Adonis group difference test was 1.0, with a *P* value of 0.001, suggesting that the intergroup differences were much greater than the intragroup differences were, thus indicating the high reliability of the test. The contributions of the first and second principal coordinates of the endophytic bacterial community were 40.48% and 34.19%, respectively. Samples of the same variety were relatively concentrated, whereas samples from different varieties were more dispersed. The resistant variety 6036 was located in the fourth quadrant, whereas varieties G3, YY87, and HD were distributed in the third, first, and third quadrants, respectively. The significant difference between the resistant variety 6036 and susceptible varieties suggests that there were considerable differences in the structure of the endophytic bacterial communities among the different resistant and susceptible varieties.

The correlation analysis of endophytic bacterial diversity in seeds of different resistant tobacco varieties with the disease index is shown in Figure 2D. The analysis results indicated a significant negative correlation (*P* < 0.05) between bacterial community diversity and the disease index from the field test, suggesting a direct relationship where greater bacterial diversity was correlated with a lower disease index.

The endophytic bacteria in seeds of different varieties were annotated to 1 domain, 1 kingdom, 8 phyla, 13 classes, 37 orders, 65 families, 106 genera, and 125 species. The community composition at the phylum level is shown in Figure 2E. At the phylum level, the endophytic bacterial communities in seeds of different varieties were similar in composition, but there were differences in relative abundance. The dominant bacterial phyla included Proteobacteria, Firmicutes, and Actinobacteria. Among these, Proteobacteria had the highest relative abundance, accounting for 94.09% (G3), 81.15% (6036), 94.94% (YY87), and 93.35% (HD); Firmicutes had relative abundances of 4.08% (G3), 9.73% (6036), 1.75% (YY87), and 2.64% (HD); and Actinobacteria accounted for 0.82% (G3), 5.98% (6036), 2.16% (YY87), and 2.31% (HD). The relative abundances of Firmicutes in the resistant varieties G3 and 6036 were significantly greater than those in the susceptible variety YY87 (2.34 and 8.46 times greater, respectively) and the susceptible variety HD (1.54 and 5.58 times greater, respectively). Additionally, the relative abundance of Actinobacteria in the resistant variety 6036 was significantly greater than those in the susceptible variety YY87 (2.40 times greater) and the susceptible variety HD (2.24 times greater).

The community composition at the genus level for the different varieties is shown in Figure 2F. The composition of endophytic bacteria at the genus level was relatively similar among different tobacco seed varieties, but there were differences in their relative abundances. The dominant genera within the seeds of various varieties primarily included *Pseudomonas, Stenotrophomonas, Paenibacillus, Allorhizobium, Pantoea, Delftia, Erwinia, Flavobacterium, Enterobacter*, and *Massilia*. Notably, the abundances of *Stenotrophomonas, Paenibacillus*, and *Allorhizobium* in the 6036 variety were significantly greater than those in the other varieties. These results indicate that there is a rich reservoir of endophytic bacterial resources within tobacco seeds, which warrants further exploration.

### 3.3 Differential microbial communities present in the endophytic bacterial community structure of seeds from tobacco varieties with varying resistance levels

**Figure 3:**
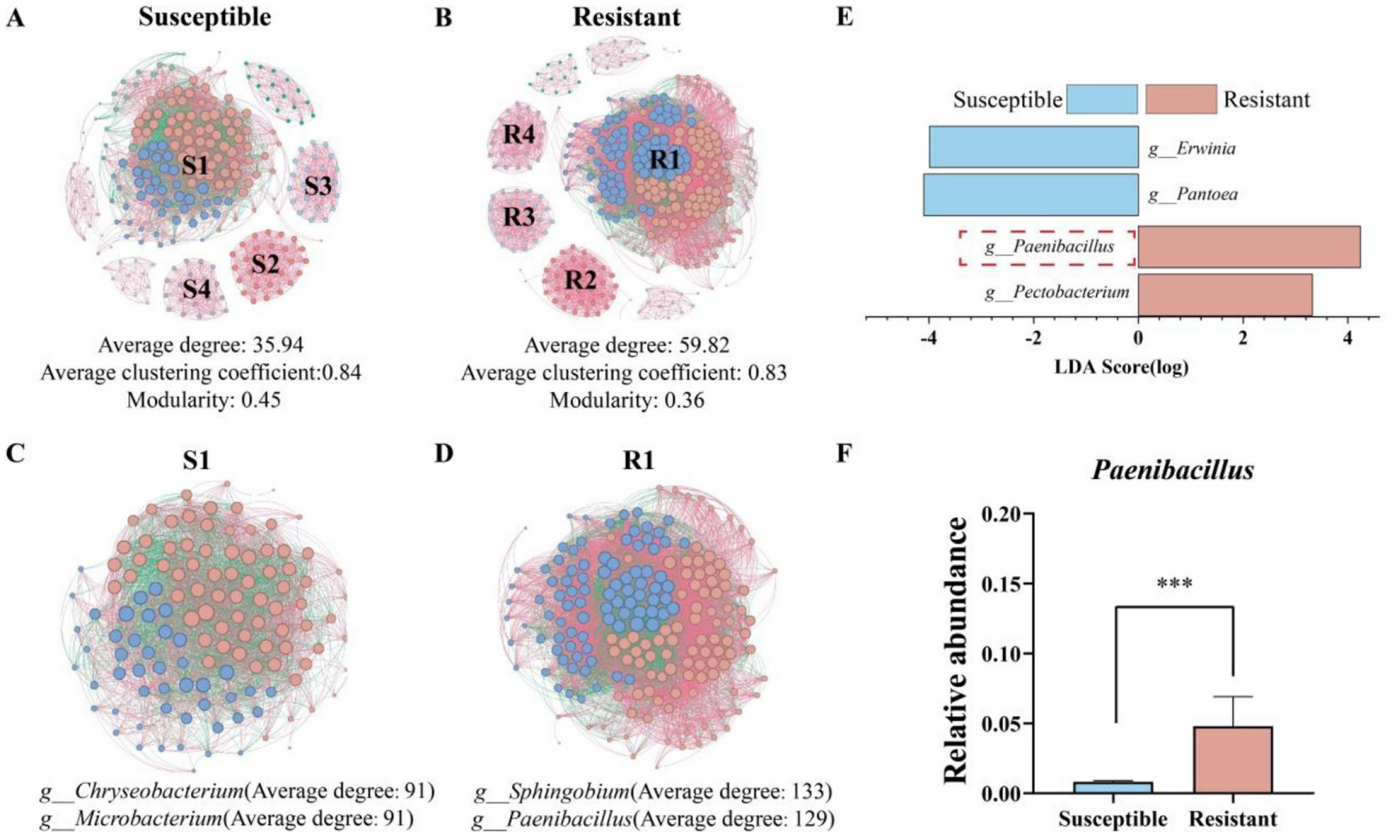
Microbial community co-occurrence networks and differential species analysis of resistant and susceptible tobacco varieties. **(A)** Co-occurrence network of the susceptible variety; **(B)** co-occurrence network of the resistant variety; **(C)** co-occurrence network of module S1 in the susceptible variety; **(D)** co-occurrence network of module R1 in the resistant variety; **(E)** LEfSe (LDA > 3) analysis of differential genera between resistant and susceptible varieties at the genus level; **(F)** comparison of the relative abundance of the differential microorganism *Paenibacillus* between resistant and susceptible varieties. The significance of the difference was determined by Unpaired t test (^***^P < 0.0001).

To analyze potential key taxa related to disease-resistant seed varieties and elucidate their interaction patterns, a co-occurrence network consisting of 500 representative bacterial ASVs was established, as illustrated in Figures 3A, B. The analysis revealed that, compared with susceptible varieties, disease-resistant varieties presented significantly greater numbers of nodes (326) and edges (9,750), with 248 nodes and 4,457 edges. The average degree of the networks for resistant varieties was also markedly greater (59.82) than that for susceptible varieties (35.94). Additionally, in the most prominent module S1 of the susceptible varieties (Figure 3C), the genera *g__Chryseobacterium* and *g__Microbacterium*, presented the highest average degrees, whereas in the predominant module R1 of the resistant varieties (Figure 3D), *g__Sphingobium* and *g__Paenibacillus* ranked first and second in average degree, respectively. To explore the key genera influencing the differences between resistant and susceptible varieties, differential genera identified by LEfSe were compared (Figure 3E). The results indicated significant enrichment of *g__Paenibacillus* (LDA = 4.24) and *Pectobacterium* (LDA = 3.32) in resistant varieties, whereas *Pantoea* (LDA = 4.10) and *Erwinia* (LDA = 3.99) were significantly enriched in susceptible varieties. Notably, *Paenibacillus* not only presented a high average degree in the co-occurrence network (degree = 129) but also presented significant differences in relative abundance at the genus level between resistant and susceptible varieties (resistant = 0.048, susceptible = 0.008), with a relative abundance that was 83.10% greater in resistant varieties (Figure 3F). Therefore, the genus *Paenibacillus* may play a crucial role in influencing the different disease resistance phenotypes exhibited by tobacco varieties.

### 3.4 Isolation and characterization of endophytic antagonistic bacteria

**Figure 4:**
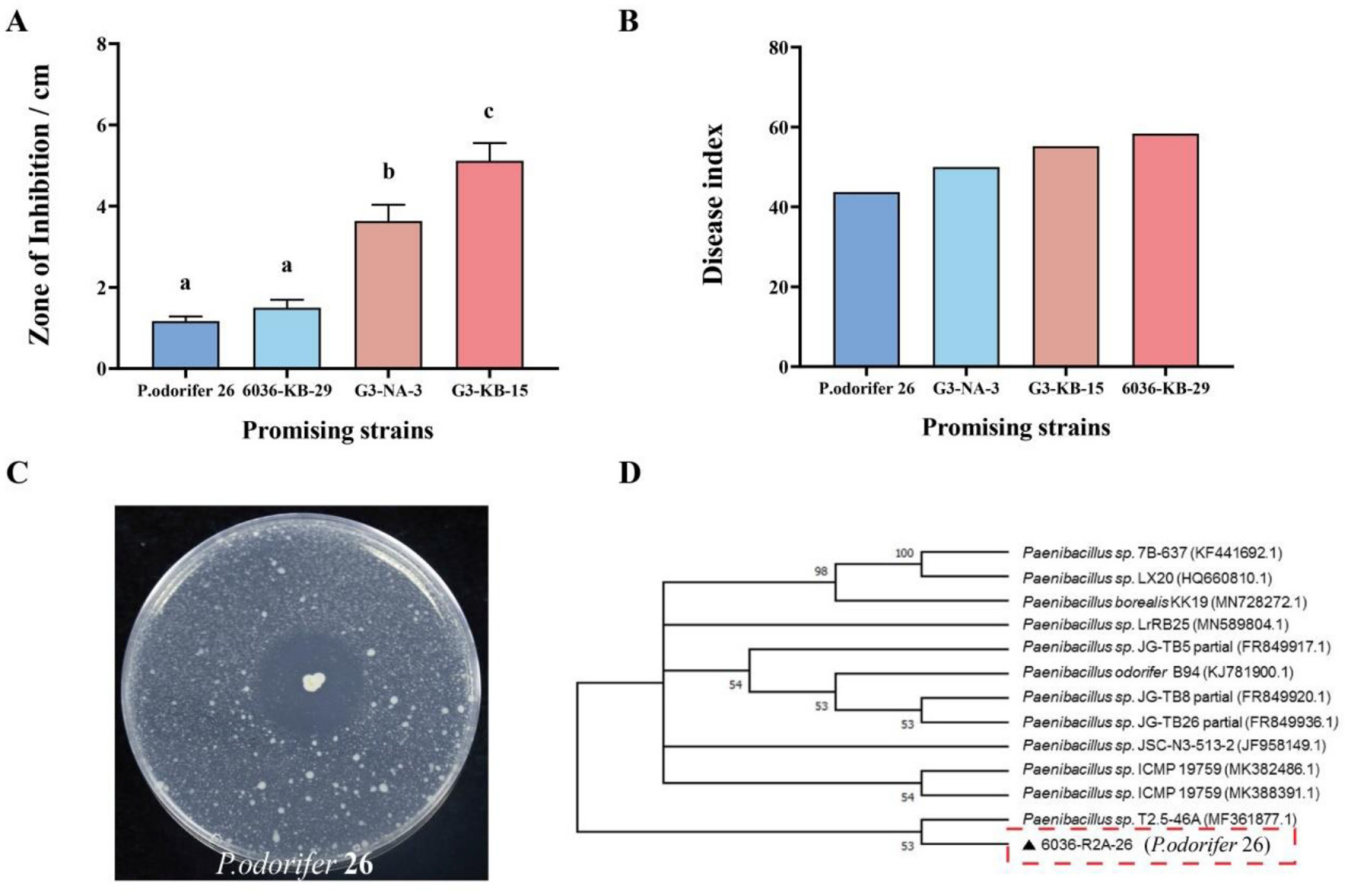
Morphological and molecular identification of strains. **(A)** Disease index; **(B)** zone of inhibition; **(C)** antagonistic plate assay of strain *P. odorifer* 26; **(D)** phylogenetic tree based on the 16S rRNA sequence of strain *P. odorifer* 26. Different letters indicate significant difference at 0.05 level among different treatments.

The diversity of endophytic bacteria in seeds is very high. Based on the phenotypic differences observed in various culture media, a total of 115 bacterial strains were isolated from variety 6036, and 112 strains were isolated from variety G3, resulting in a total of 227 endophytic bacteria obtained from all resistant seed varieties (Supplementary Figure S2). We evaluated the antibacterial activity of these endophytic bacteria from resistant seed varieties using plate assays. Potential biocontrol strains subsequently underwent preliminary pot experiments, as illustrated in Figures 4A, B. The results showed that strains exhibiting larger inhibition zones in plate assays did not necessarily display good biocontrol efficacy under greenhouse conditions. This finding indicates that there was no positive correlation between biocontrol effectiveness and the size of the inhibition zone. Through multiple repeated pot experiments, we ultimately selected one endophytic antagonistic bacterium (6036-R2A-26) that demonstrated better and more stable performance for subsequent experiments.

The 6036-R2A-26 strain, as shown in Figure 4C, is white, orbicular, opaque, and contains elliptical spores within expanded cysts. The target gene sequences obtained from different endophytic antagonist bacteria were analyzed by 16S rRNA sequence analysis and compared using BLAST in the GenBank database, leading to the construction of a phylogenetic tree (Figure 4D). Based on the constructed phylogeny, 6036-R2A-26 strain was identified as *Paenibacillus odorifer* (deposited at the China General Microbiological Culture Collection Center, Wuhan; deposition date: June 7, 2022; accession number: CCTCC NO: M 2022821). This strain will be referred to as *P. odorifer* 26 for subsequent reference.

### 3.5 The antagonistic strains effectively control tobacco diseases and promote growth

#### 3.5.1 Antagonistic bacteria can effectively control the occurrence of BWD

**Figure 5:**
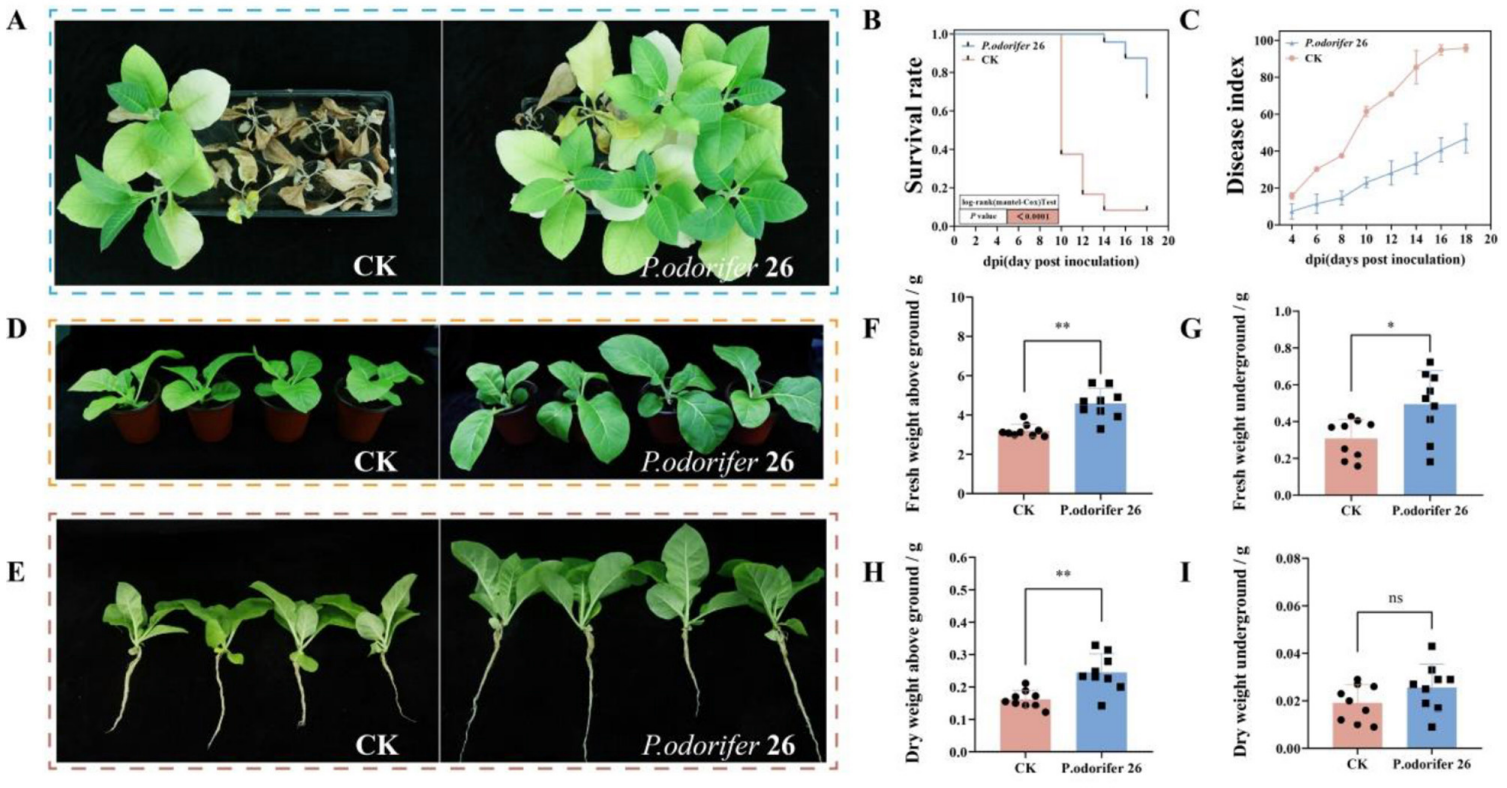
Results of potted plant experiments with two strains of antagonistic bacteria. **(A)** Disease incidence of tobacco plants after inoculation for 20 days; **(B)** survival curve; **(C)** disease index; **(D)** tobacco plant growth; **(E)** tobacco root growth; **(F)** aboveground fresh weight; **(G)** underground fresh weight; **(H)** aboveground dry weight; **(I)** underground dry weight. The significance of the difference was determined by Unpaired t test (^**^P < 0.001; ^*^P < 0.05; ns: not significant).

The efficacy of the bacterium P. odorifer 26 in controlling BWD was evaluated, with the results recorded 20 days postinoculation and presented in Figure 5A. Five days after inoculation with the wilt pathogen, all the treatment groups began to show symptoms, and the disease index in each treatment group gradually increased over time, whereas the survival rate decreased. Figure 5B shows the survival curves of the treated tobacco plants after inoculation; by the 14th day postinoculation, the survival rate of the control group (CK) was only 10%, whereas the survival rate of the P. odorifer 26 group remained above 60% by the 18th day postinoculation. Moreover, as shown in Figure 5C, the disease index in the control group (CK) consistently remained greater than that in the treatment group with the P. odorifer 26 bacterium. The data from the 18th day indicated that the disease index for the P. odorifer 26 treatment was 48.95, whereas it was 95.83 for the CK group, representing a significant reduction in the disease index of 51.08% with the P. odorifer 26 treatment. These results demonstrate that this strain is effective in controlling BWD and suggest broad application prospects.

#### 3.5.2 Antagonistic bacteria can effectively promote the early growth and rapid development of tobacco

The effects of P. odorifer 26 bacterial treatment on the biomass of tobacco seedlings are shown in Figures 5D–I. Compared with those in the CK treatment, the above-ground fresh weight, underground fresh weight and above-ground dry weight significantly increased by 30.26%, 37.75%, and 33.97%, respectively, in the tobacco treated with P. odorifer 26. These results demonstrate that continuous root irrigation with P. odorifer 26 bacterial fermentation mixture can effectively promote tobacco growth at the tobacco seedling stage.

## 4 Discussion

Plant endophytes are a group of microorganisms that reside within the tissues of the host plant without causing any discernible symptoms (Félix et al., 2023). A growing body of research is now exploring the potential of endophytes as a means of enhancing plant health (Daria et al., 2017; Ghosh et al., 2021). Despite studies demonstrating that beneficial endophytic microorganisms are enriched in both diseased roots and stems after infection with *R. solanacearum*, the inoculation of soil with the beneficial root-derived endophyte *Burkholderia cepacia* has been shown to significantly enhance the defense system of tobacco in the presence of pathogens, thereby reducing disease incidence (Tao et al., 2024). Our field investigation revealed significant differences in resistance to BWD among different tobacco varieties. Generally, seeds carry the genetic information underlying the varieties of which they are composed; therefore, it was hypothesized that this phenomenon may be associated with the seed endophytes. To confirm this correlation, we conducted a high-throughput sequencing analysis of diverse tobacco seed endophyte varieties. First, based on phenotypic assessments, we identified 6036 and G3 as resistant varieties, and the results from pot experiments confirmed these findings. Similarly, YY87 was recognized as a susceptible variety in a study by Li et al. (2021). Preliminary analysis of the structure of the seed endophytic bacterial communities revealed that the resistant variety 6036 contained a more diverse and abundant bacterial community, with both its Chao1 and Shannon indices significantly higher than those of the other varieties (*P* < 0.05). This variety also presented unique community structural characteristics, with higher abundances of *Stenotrophomonas, Paenibacillus*, and *Allorhizobium* than the other varieties. Notably, bacteria within the *Stenotrophomonas* genus have been confirmed to degrade phenolic allelochemicals (PAs), which can help control BWD (Chang et al., 2022). Furthermore, inoculation with *Paenibacillus polymyxa* promoted tobacco growth by inducing the expression of plant hormone-related genes such as auxins and cytokinins (Liu et al., 2020). Interestingly, this study also revealed that the strain *Paenibacillus* polymyxa upregulated the expression of transcription factors related to stress resistance in tobacco plants. The enrichment of these functional groups may confer special disease resistance capabilities to the variety 6036. The complexity of the microbial network is closely intertwined with the stability of community structure and soil functionality (Zhang et al., 2022). The nodes and edges in the resistant varieties were significantly greater than those in the susceptible varieties, a characteristic that may contribute to their increased adaptability. Further analysis revealed that *g_Sphingobium* and *g_Paenibacillus* play critical roles as network hubs within the seed endophytic bacterial communities. Interestingly, *g__Paenibacillus* was identified as a key differentiating bacterium between resistant and susceptible varieties. *Paenibacillus* occupies niches both near and inside plants, a trait that has persisted since the evolution of early land plants (Langendries and Goormachtig, 2021). Scanning electron microscopy has confirmed its ability to colonize cucumber seeds and root systems (Park et al., 2004), and it has been found to inhabit the stem and leaf tissues of cucumbers and corn (Hao and Chen, 2017). Furthermore, some studies suggest that *Paenibacillus* can coexist with the genetic evolution of corn seeds (Johnston-Monje and Raizada, 2011), indicating significant biocontrol potential. In addition, *Paenibacillus* has been frequently reported for use in the biocontrol of BWD (Zhao et al., 2020). We isolated an antagonist strain, *Paenibacillus odorifer* (*P. odorifer 26*), from the seeds of resistant tobacco varieties. When we tested its efficacy in controlling BWD, we observed an interesting phenomenon: in the agar diffusion assay, the inhibition zone produced by *P. odorifer* 26 when confronted with the wilt pathogen was not particularly pronounced. However, in the pot experiments, it significantly reduced the disease index. This led us to speculate that the biocontrol effect may not correlate positively with the size of the inhibition zone in plate assays. Furthermore, *P. odorifer* 26 has also demonstrated substantial potential for promoting tobacco growth. This potential can be attributed to the beneficial and harmonious symbiotic relationships within the seed endophytic bacterial community, growth-promoting characteristics and antibacterial activity. These findings underscore the superiority of seed endophytic microorganisms as potential agents for enhancing agricultural ecosystem productivity. A promising area for future research is whether this potential can be retained across multiple generations of seeds, given their vertical transmission characteristics.

## 5 Conclusion

Taken together, the results of this study revealed differences in the structural characteristics of endophytic bacterial communities between resistant and susceptible tobacco varieties, with groups such as *Paenibacillus* potentially playing significant roles in resisting BWD. Furthermore, a bacterium identified as *Paenibacillus odorifer* was isolated from the seeds of the resistant variety 6036, which demonstrated effective control over BWD, along with positive growth-promoting effects. This bacterium can be considered a promising novel microbial antagonist for the management of BWD. The potential of this strain can be attributed to the beneficial and harmonious symbiotic relationships within the endophytic bacterial community, as well as its growth-promoting characteristics and antibacterial activity. These findings highlight the superiority of seed endophytic microorganisms. In the context of declining plant disease resistance and the spread of bacterial wilt, core endophytic microorganisms in seeds may emerge as a viable option for enhancing the productivity of agricultural ecosystems.

## Data Availability

The raw sequencing data have been deposited in the NCBI Sequence Read Archive (SRA) database under the accession number PRJNA1196667. It can be accessed at: https://www.ncbi.nlm.nih.gov/sra/PRJNA1196667.
