# Supplementary material for: Superiority of native seed core microbiomes in the suppression of bacterial wilt disease
Source: Front Microbiol. 2025 Jan 15;15:1506059. doi: 10.3389/fmicb.2024.1506059 (PMC11778171; doi:10.3389/fmicb.2024.1506059)
Supplement: Supplementary file 1 [file Data_Sheet_1.pdf]

Supporting information

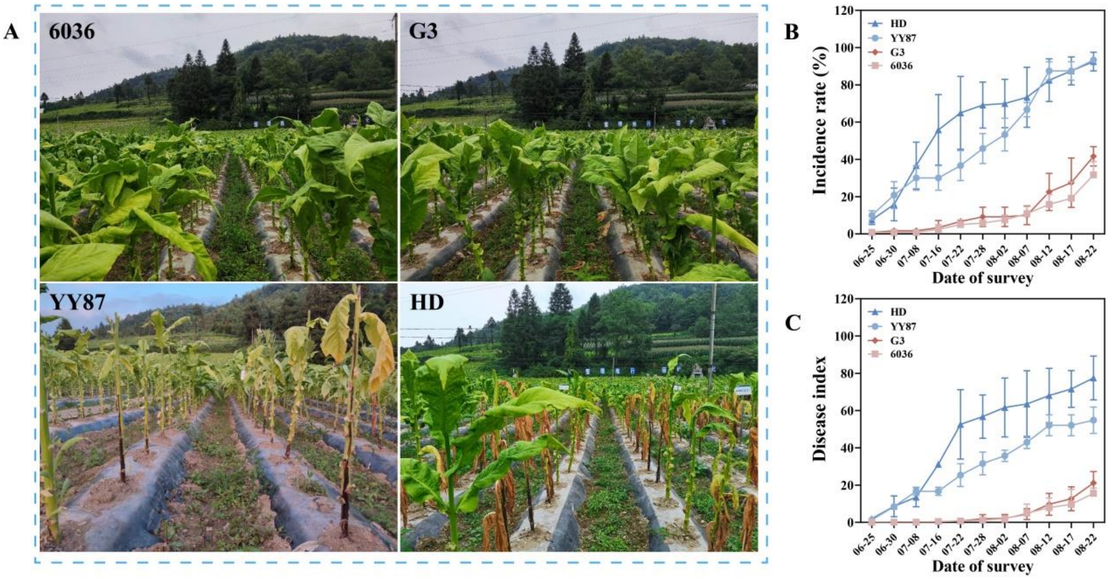

Figure 1 Validation of disease resistance in different tobacco varieties through field trials. (A) Disease incidence in different tobacco varieties during the mid to late growth stage; (B) Incidence rate of different tobacco varieties from early to mid-stage disease progression; (C) Disease index of different tobacco varieties from early to mid-stage disease progression.

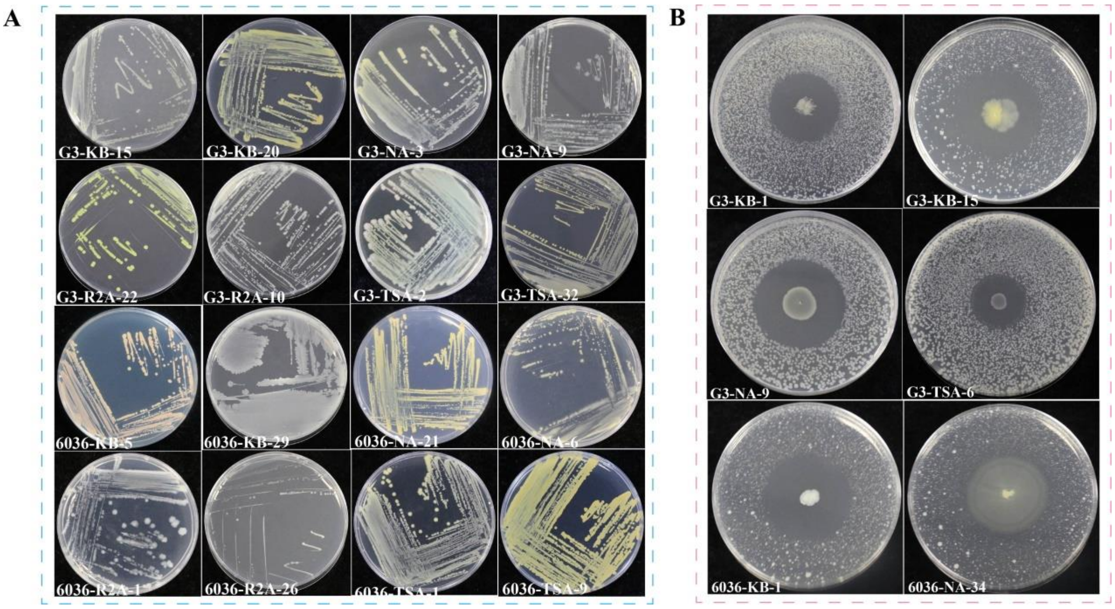

Figure 2 Culturable bacteria isolated from resistant tobacco varieties 6036 and G3. (A) Morphological images of different bacterial morphologies on various media (partial); (B) Bacteria with notable antagonistic effects against *R. solanacearum* (partial).
